# Doctors’, nurses’ and clinical associates’ understanding of emergency care practitioners

**DOI:** 10.4102/hsag.v26i0.1523

**Published:** 2021-03-11

**Authors:** Craig Vincent-Lambert, Dirk Kotzé

**Affiliations:** 1 Department of Emergency Medical Care, Faculty of Health Sciences, University of Johannesburg, Johannesburg, South Africa

**Keywords:** emergency department, inter-professional education, inter-professional collaboration, patient handover, teamwork

## Abstract

**Background:**

Healthcare professionals’ understanding of the knowledge, skills and training of their counterparts from other disciplines cultivates appreciation and respect within the workplace. This, in turn, results in better teamwork and improved patient care. Emergency departments are places where emergency care practitioners (ECPs) engage with doctors, nurses and clinical associates. Whilst the importance of inter-professional communication and teamwork between in-hospital professionals and pre-hospital emergency care providers is acknowledged, no literature could be found describing exactly how much these in-hospital professionals understand about the training and capabilities of their ECP colleagues.

**Aim:**

The aim of this study was to assess the level of understanding that prospective doctors, nurses and clinical associates have regarding the training and capabilities of ECPs.

**Setting:**

The research was conducted in Johannesburg, South Africa, at two universities.

**Methods:**

Seventy-seven participants completed a purpose-designed questionnaire assessing their understanding regarding the education and clinical capabilities of ECPs.

**Results:**

In total, 64% of participants demonstrated a poor understanding of the level of education and clinical capabilities of ECPs. The remaining 36% showed only moderate levels of understanding.

**Conclusion:**

Medical, nursing and clinical associate graduates have a generally poor understanding of the education and clinical capabilities of their ECP colleagues who practise predominantly in the pre-hospital environment. This lack of understanding can become a barrier to effective communication between ECPs and in-hospital staff during patient handover in emergency departments.

**Contribution:**

This research highlights a lack of understanding about the role and function of South African ECPs as pre-hospital emergency care providers and the need for more effective inter-professional education.

## Introduction

Teamwork, inter-professional education (IPE) and inter-professional collaboration (IPC) may address many of the challenges faced by healthcare systems (Reeves et al. 2017; World Health Organization [WHO] 2010, 2013). Consequently, the widespread use and promotion of IPE and IPC have been advocated as a way of cultivating respect amongst healthcare professionals from different disciplines by promoting patient-centred practices (Reeves et al. 2008; WHO 2010).

In emergency care and primary healthcare settings, effective collaborative practice has been found to result in improved access to, and coordination of, health services, increased acceptance of care, and higher levels of patient satisfaction. In addition, effective collaborative practices have been shown to decrease patient complications, reduce the length of hospital stays and decrease the cost of care. Furthermore, effective teamwork and collaborative practice also hold additional benefits for employers by reducing conflict and tension amongst colleagues, decreasing staff turnover and increasing job satisfaction (Carney et al. 2019; Lemieux-Charles & McGuire 2006; Mickan 2005; Morley & Cashell 2017; WHO 2010).

South African emergency care practitioners (ECPs) engage with other members of the healthcare team during patient handover in the emergency department (ED) (Johnston, MacQuarrie & Rae 2014). Emergency care practitioners complete a 4-year degree in emergency medical care and rescue at an accredited higher education institution (Makkink et al. 2019). After completion of the degree, ECPs register with the Health Professions Council of South Africa (HPCSA) and are able to provide emergency and critical care in a wide range of settings, most commonly the pre-hospital setting. Emergency care practitioners are also able to function as part of rescue teams specialising in a number of rescue disciplines (Faculty of Emergency Medical Care: University of Johannesburg 2020).

During handovers ECPs will commonly interact with three different types of healthcare professionals: medical doctors, nurses and clinical associates (Donaldson 2008; Doherty, Couper & Fonn 2012; Johnston et al. 2014; O’Daniel & Rosenstein 2008). Whilst the medical and nursing professions have a long history, and as such, are generally well recognised and understood, clinical associates are a relatively new cadre of South African healthcare professional introduced in 2008 by the National Department of Health. Their introduction was aimed at addressing a shortage of healthcare workers, specifically doctors. Clinical associates function in a variety of healthcare settings, including community clinics and district hospitals. Clinical associates register with the HPCSA after completing a 3-year Bachelor of Clinical Medical Practice (BCMP).

During handover, reliable and accurate information must be transferred between ECPs and their counterparts in the ED to maintain continuity of care and ensure patient safety (Calleja, Aitken & Cooke 2011). Poor handover between emergency medical services (EMS) providers and in-hospital staff in the ED has been identified as one of the major causes of medical error, and a lack of effective IPC has been cited as a contributing factor (Johnston et al. 2014; Siemsen et al. 2012).

The implementation of effective IPC has, however, proved to be challenging, and the literature identifies a number of barriers to effective IPC (Courtenay, Nancarrow & Dawson 2013; Hall 2005; O’Daniel & Rosenstein 2008). These barriers include a lack of understanding and appreciation of the knowledge, skills and training of other members of the team (O’Daniel & Rosenstein 2008; Suter et al. 2009). Such barriers not only inhibit the implementation of collaborative practice but also the efforts to teach healthcare professionals’ understanding of the skills and knowledge required to work in a collaborative environment.

Understanding on the part of healthcare providers about the training and abilities of other practitioners represents an important component of successful IPE and IPC (Bollen et al. 2019; Hepp et al. 2014; Karam et al. 2018; Reeves et al. 2014). Authors of IPC frameworks often agree that a clear understanding of professional roles and scope of practice is required for successful IPC. The need for professionals to understand the training, education and clinical paradigm of other professionals is also frequently mentioned (Gaboury et al. 2009; Karam et al. 2018). However, most studies focus on IPC and the understanding that exists between in-hospital healthcare professionals such as doctors, nurses and radiographers. No literature could be found describing exactly how much doctors, nurses and clinical associates understand about their ECP colleagues. Our study therefore aimed to assess the level of understanding that prospective doctors, nurses and clinical associates have regarding the training and capabilities of ECPs.

## Methods

For our study we used non-probability convenience sampling as it was well suited for this descriptive/exploratory work and given the need for ease of access to participants and the willingness of the individuals/groups to participate.

As mentioned above, during their daily activities, ECPs commonly encounter three different healthcare professionals: medical doctors, nurses and clinical associates. The population therefore included students enrolled in the Bachelor of Medicine and Bachelor of Surgery (MBBCh) degree and the BCMP degree at university 1 and the Bachelor of Nursing (BCur Nursing) degree students from university 2. This population was chosen as the students in these programmes will eventually qualify and become part of the in-hospital staff, who will receive patients and handovers from ECPs. In addition, we assumed that throughout their studies these students would have encountered ECPs during their clinical work and should therefore have been able to provide some comment relating to their understanding about what an ECP is and does.

In total, 77 participants (33 nursing, 25 medical and 19 clinical associate students) from two South African universities completed a pre-piloted, purpose-designed survey questionnaire. The participants were all final-year students enrolled in the MBBCh, BCur or BCMP programmes at their respective institutions.

The self-developed questionnaire composed of 30 carefully constructed questions that focused on exploring participants’ understanding relating to the level and duration of education and training of an ECP, including selected clinical capabilities and scope of practice. The questions were all closed ended and had pre-set response options with only one correct answer. The participant responses to each of the questions could thus be classified as either correct or incorrect.

A pilot study was performed to assess and improve the face validity and reliability of the self-developed questionnaire. The pilot study was performed on a small group of volunteers consisting of two final-year students from each of the three health professions that made up the sample. Steps used to perform the pilot study included the following:

- The researcher presented the questions to the pilot participants in the same way as they would have appeared to participants in the actual study.
- Feedback was obtained from the pilot participants to identify if there were difficult questions and/or ambiguities.
- The researcher also recorded the time taken by the pilot participants to complete the questionnaire to decide if it was reasonable.
- Following feedback, any ambiguous or unnecessary questions were removed.
- In addition, the researcher focused on establishing that the answers provided could be interpreted in terms of the information that was required.
- The researcher then reworded or rescaled any questions that were not answered as expected.

Responses to the questions were analysed using simple descriptive statistics. For each question, the number and percentage of correct responses was calculated for each of the three disciplines separately as well for the overall group performance. To gauge the level of understanding across all 30 questions, a score of one point was allocated for each correct response. This total score, out of 30, was then converted into a percentage.

We then used the overall percentages to further categorise levels of understanding as ‘poor’ for respondents who scored 50% or less, ‘moderate’ for those who scored between 51% and 74%, and ‘good’ for those who scored 75% and above. The sample means were calculated, allowing for a comparison of scores for each of the three cadres of participants.

## Ethical considerations

Ethical clearance for this study was granted by the University of Johannesburg, Faculty of Health Sciences Research Ethics Committee (NHREC Registration REC 241112-035); the ethical clearance number was REC-01-47-2018.

## Results

The responses for the 30 questions focusing on the level of education and clinical capabilities of ECPs are summarised in Tables 1–3.

**TABLE 1:** Summary of responses (questions 1–9).

Question	Options†	Number of correct responses							
		BCur		MBBCh		BCMP		Total	
		*n*	*%*	*n*	*%*	*n*	*%*	*n*	*%*
1. What is the highest level of care that emergency care practitioners are trained to provide?	Basic life support Intermediate life support **Advanced life support** I do not know	**12/33**	**36**	**16/25**	**64**	**12/19**	**63**	**40/77**	**52**
2. Are emergency care practitioners trained to be supervised practitioners or independent practitioners?	Supervised practitioners **Independent practitioners** I don’t know	**29/33**	**88**	**14/25**	**56**	**11/19**	**58**	**54/77**	**70**
3. What is the duration of training of emergency care practitioners?	9 months 1 year 2 years 3 years **4 years** I don’t know	**23/33**	**70**	**9/25**	**36**	**6/19**	**32**	**38/77**	**49**
4. Is the training of emergency care practitioners regulated by some form of regulatory body?	**Yes** No I don’t know	**24/33**	**73**	**12/25**	**48**	**16/19**	**84**	**52/77**	**68**
5. Who is responsible for the regulation of the training of emergency care practitioners?	**HPCSA** SANC ECSSA None of the above	**6/33**	**18**	**9/25**	**36**	**6/19**	**32**	**21/77**	**27**
6. ECPs are able to identify a patient with a compromised airway.	**Yes** No Unsure	**33/33**	**100**	**24/25**	**96**	**18/19**	**95**	**75/77**	**97**
7. ECPs are able to perform rapid sequence intubation (RSI) in the pre-hospital environment.	**Yes** No Unsure	**22/33**	**67**	**15/25**	**60**	**12/19**	**63**	**49/77**	**64**
8. ECPs are able to perform a surgical cricothyrotomy in the pre-hospital environment.	**Yes** No Unsure	**4/33**	**12**	**8/25**	**32**	**3/19**	**16**	**15/77**	**19**
9. ECPs are able to perform external jugular venous cannulation in the pre-hospital environment for the administration of fluids or drugs.	**Yes** No Unsure	**21/33**	**64**	**11/25**	**44**	**10/19**	**53**	**42/77**	**55**

BCur Nursing, Bachelor of Nursing; MBBCh, Bachelor of Medicine and Bachelor of Surgery; BCMP, Bachelor of Clinical Medical Practice; ECP, emergency care practitioner; HPCSA, Health Professions Council of South Africa; SANC, South African Nursing Council; ECSSA, Emergency Care Society of South Africa.

† , Bold indicates the correct option.

**TABLE 2:** Summary of responses (questions 10–19).

Question	Options†	Number of correct responses							
		BCur		MBBCh		BCMP		Total	
		*n*	*%*	*n*	*%*	*n*	*%*	*n*	*%*
10. ECPs are able to perform intra-osseous catheter placement in the pre-hospital environment for the administration of fluids or drugs.	**Yes** No Unsure	**9/33**	**27**	**11/25**	**44**	**5/19**	**26**	**25/77**	**32**
11. ECPs are able to place an arterial line in the pre-hospital environment for the administration of fluids and drugs.	Yes **No** Unsure	**4/33**	**12**	**10/25**	**40**	**1/19**	**5**	**15/77**	**19**
12. ECPs are able to perform 12-Lead electrocardiograms (ECGs) and diagnose ST elevation myocardial infarction (STEMI) in the pre-hospital environment.	**Yes** No Unsure	**21/33**	**64**	**17/25**	**68**	**7/19**	**37**	**45/77**	**58**
13. ECPs are able to measure and interpret cardiac enzymes in the pre-hospital environment.	Yes **No** Unsure	**8/33**	**24**	**13/25**	**52**	**4/19**	**21**	**25/77**	**32**
14. ECPs are able to perform thrombolysis in the pre-hospital environment.	**Yes** No Unsure	**6/33**	**18**	**5/25**	**20**	**6/19**	**32**	**17/77**	**22**
15. ECPs are able to identify septic patients in the pre-hospital environment.	**Yes** No Unsure	**27/33**	**82**	**19/25**	**76**	**14/19**	**74**	**60/77**	**78**
16. ECPs are able to initiate an adrenaline infusion to provide haemodynamic support to septic patients in the pre-hospital environment.	**Yes** No Unsure	**20/33**	**61**	**19/25**	**76**	**16/19**	**84**	**55/77**	**71**
17. ECPs are able to initiate empiric broad-spectrum antibiotic therapy in the pre-hospital environment.	Yes **No** Unsure	**9/33**	**27**	**8/25**	**32**	**5/19**	**26**	**22/77**	**29**
18. ECPs are able to administer Magnesium Sulphate for the management of eclampsia in the pre-hospital environment.	**Yes** No Unsure	**21/33**	**64**	**7/25**	**28**	**6/19**	**32**	**34/77**	**44**
19. ECPs are able to identify and manage a breech delivery in the pre-hospital environment.	**Yes** No Unsure	**16/33**	**48**	**11/25**	**44**	**11/19**	**58**	**38/77**	**49**

BCur Nursing, Bachelor of Nursing; MBBCh, Bachelor of Medicine and Bachelor of Surgery; BCMP, Bachelor of Clinical Medical Practice; ECP, emergency care practitioner; ST, segment.

† , Bold indicates the correct option.

**TABLE 3:** Summary of responses (questions 20–30).

Question	Options†	Number of correct responses							
		BCur		MBBCh		BCMP		Total	
		*n*	*%*	*n*	*%*	*n*	*%*	*n*	*%*
20. ECPs are able to delay labour through the administration of tocolytics.	**Yes** No Unsure	**10/33**	**30**	**1/25**	**4**	**4/19**	**21**	**15/77**	**19**
21. ECPs are able to measure intracranial pressure in the pre-hospital environment.	Yes **No** Unsure	**4/33**	**12**	**18/25**	**72**	**8/19**	**42**	**30/77**	**39**
22. ECPs are able to administer mannitol to patients with traumatic brain injuries.	Yes **No** Unsure	**7/33**	**21**	**8/25**	**32**	**3/19**	**16**	**18/77**	**23**
23. ECPs are able to administer paralytics to prevent reflexes that could have a detrimental effect in patients with traumatic brain injuries (TBI).	**Yes** No Unsure	**12/33**	**36**	**11/25**	**44**	**11/19**	**58**	**34/77**	**44**
24. ECPs are able to perform needle thoracentesis in patients with a tension pneumothorax.	**Yes** No Unsure	**10/33**	**30**	**17/25**	**68**	**15/19**	**79**	**42/77**	**55**
25. ECPs are able to insert an intercostal drain for the management of a tension pneumothorax in the pre-hospital environment.	Yes **No** Unsure	**7/33**	**21**	**14/25**	**56**	**9/19**	**47**	**30/77**	**39**
26. ECPs are able to perform blood transfusions for the management of severe bleeding in the pre-hospital environment.	Yes **No** Unsure	**9/33**	**27**	**11/25**	**44**	**6/19**	**32**	**26/77**	**34**
27. ECPs are able to manage bradycardias with agents such as adrenaline and atropine.	**Yes** No Unsure	**24/33**	**73**	**18/25**	**72**	**16/19**	**84**	**58/77**	**75**
28. ECPs are able to manage bradycardias with transcutaneous pacing.	**Yes** No Unsure	**14/33**	**42**	**10/25**	**40**	**9/19**	**47**	**33/77**	**43**
29. ECPs are able to manage tachycardias with agents such as adenosine and amiodarone.	**Yes** No Unsure	**19/33**	**58**	**12/25**	**48**	**10/19**	**53**	**41/77**	**53**
30. ECPs are able to manage tachycardias with non-pharmacologic measures such as the modified Valsalva manoeuvre and synchronised cardioversion.	**Yes** No Unsure	**20/33**	**61**	**16/25**	**64**	**14/19**	**74**	**50/77**	**65**

BCur Nursing, Bachelor of Nursing; MBBCh, Bachelor of Medicine and Bachelor of Surgery; BCMP, Bachelor of Clinical Medical Practice; ECP, emergency care practitioner.

† , Bold indicates the correct option.

As can be seen, the first five questions focused on exploring participants’ understanding of the education and training of ECPs. Analysis of responses showed a mean score for all participants across this section of 2.7/5 (54%). The nursing students had the highest level of understanding about the training of ECPs, with a mean score of 3/5 (60%). Bachelor of Clinical Medical Practice students followed closely with a mean score of 2.6/5 (54%), whilst MBBCh students came third with a mean score of 2.3/5 (47%).

Analysis of the remaining 25 questions, which focus on exploring understanding relating to aspects of the clinical competence and scope of practice for an ECP, delivered a mean score for the entire group of 11.6/25 (46%). Nursing students demonstrated the lowest level of understanding about the clinical capabilities of ECPs, with a mean score of 10.6/25 (42%). Bachelor of Medicine and Bachelor of Surgery students had the highest level of understanding, with a mean score of 12.7/25 (51%), whilst the BCMP students were ranked second with a mean score of 11.7 (47%).

In terms of overall understanding regarding the training and abilities of ECPs, 49/77 (64%) of our participants scored 50% or less, thus demonstrating a poor level of understanding. A total of 28/77 (36%) of the participants had a moderate level of understanding, scoring between 51% and 74%, and not one of the participants demonstrated a good level of understanding by scoring more than 74% overall.

**FIGURE 1:**
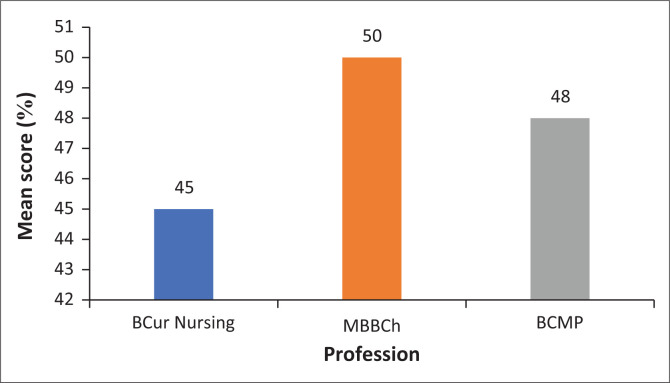
Mean score per profession. BCur Nursing, Bachelor of Nursing; MBBCh, Bachelor of Medicine and Bachelor of Surgery; BCMP, Bachelor of Clinical Medical Practice.

To compare the levels of understanding across the three different disciplines, the mean overall scores were calculated. In this regard, the nursing students had the lowest level of understanding about the training and abilities of ECPs, with a mean overall score of 13.6/30 (45%). Bachelor of Medicine and Bachelor of Surgery students, on the contrary, had the best level of understanding, with a mean overall score of 15.1/30 (50%). Finally, BCMP students had a mean overall score of 14.4/30 (48%). These results are summarised in Figure 1.

## Discussion

Our study revealed that none of the soon-to-graduate nurses, doctors and clinical associates surveyed had a good understanding of the training and clinical capabilities of their ECP counterparts. The majority (64%) demonstrated a poor level of understanding, and the remaining 36% showed only moderate levels of understanding of ECPs as members of the healthcare team.

These findings are not entirely unexpected and may be attributed to the fact that the 4-year professional bachelor’s degree in emergency medical care that produces ECPs is a relatively new qualification on the African EMS landscape, and South African ECPs are rarely involved in IPE. This is supported by the findings of other studies, which describe the most common professions involved in IPE, and these do not include ECPs (Hammick et al. 2007; Maree & Wyk 2016; WHO 2010).

A second possible reason for the poor understanding our participants had regarding ECPs is that there are so many different pre-hospital qualifications in South Africa. The historically chaotic state of EMS training in the country means that South Africa still sits with a legacy of multiple qualifications and different variations thereof, all producing different cadres of pre-hospital emergency care providers. Variations in the level of training and scope of practice linked to historical and current pre-hospital qualifications are significant; this continues to lead to confusion both inside and outside the industry (Nielsen et al. 2012).

The education and training of healthcare providers form their specific professional identities and culture (Pullon 2008). Understanding of the education and training of other healthcare professionals therefore plays a role in promoting an understanding of the values and beliefs held by different professionals whilst also addressing common misconceptions or stereotypes. Consequently, when mutual trust and respect are built, IPC is also advanced (Bartunek 2011; MacDonald et al. 2010). The findings of this study are seen to be interesting and important, as the low levels of understanding displayed by participants in this study have implications for both IPC and IPE. This is because studies cite a student’s lack of understanding of the training of other students as a cause of confusion and role blurring. This may result in tension and conflict amongst professionals post-graduation, which ultimately inhibits effective IPE and IPC in the workplace (Lachmann et al. 2013).

The responses also revealed that our participants understood less about the clinical capabilities of ECPs than they did about their education and training. The recent implementation of new clinical practice guidelines for South African pre-hospital providers and the changes made to the scope of practice of ECPs described therein may also have contributed to this finding. Regardless of the reason, the lack of understanding our participants demonstrated about the abilities of ECPs represents a challenge because a lack of inter-professional understanding with regard to the scope of practice between professions is acknowledged as a barrier to effective IPE and IPC (MacDonald et al. 2010).

We feel that there is a need for increased IPE and further research into solutions that seek to improve the level of understanding of members of the healthcare team working in EDs regarding the training and clinical capabilities of all cadres of pre-hospital caregiver, including ECPs. We further argue that meaningful IPE should be more than students from different healthcare professions simply sitting in the same class together. Rather, IPE should involve medical, nursing, clinical associate and ECP students being purposefully placed in contexts where they are expected to work together to solve problems and provide team-based care during both simulated and authentic clinical learning experiences.

### Study limitations

An acknowledged limitation of this study was the use of a non-validated questionnaire for data collection. This was, however, unavoidable because at the time of the study no validated questionnaire could be identified to address the proposed area of inquiry. Another important limitation of this study was the sample size. Our goal of 90 participants with an even spread of 30 participants per cadre was not reached therefore; although we came close, we did not have a perfectly even distribution of participants per cadre. In our study, we focused on senior students from three different disciplines, who we thought would have had sufficient prior interactions with ECPs (both in the classroom and during clinic learning placements) to form an understanding regarding their training and capabilities. However, we acknowledge that differences in our participants’ education and training programmes mean that they may not all have had the same exposure to ECPs. This may explain (in part) the differences noted in levels of understanding across the three disciplines. Finally, the usual limitations of survey research, such as the potential for dishonesty, a lack of conscientious responses, or differences in understanding and interpretation, could have influenced our results.

## Conclusion

In conclusion, our study found that prospective in-hospital professionals do not have a deep understanding of the training and clinical capabilities of their South African ECP colleagues. This may be for a variety of reasons, including the diverse nature of the South African EMS system, the existence of unique professional cultures and the formation of profession-based silos.

Regardless of the reasons, this lack of understanding has significant implications for IPC amongst these in-hospital professionals and pre-hospital ECPs when they engage in the workplace. Further research needs to be conducted to identify solutions that seek to improve the level of understanding of members of the healthcare team working inside EDs about the training and clinical capabilities of pre-hospital ECPs.
